# Balloon compression-induced spinal cord injury in canines: a large animal model for spinal cord injury research

**DOI:** 10.25122/jml-2023-0531

**Published:** 2024-05

**Authors:** Yudha Mathan Sakti, Emir Riandika Samyudia, Deas Makalingga Emiri, Teguh Aryandono, Rahadyan Magetsari, Rusdy Ghazali Malueka, Ery Kus Dwianingsih

**Affiliations:** 1 Orthopedics and Traumatology Department, Dr. Sardjito General Hospital, Faculty of Medicine, Public Health and Nursing, Universitas Gadjah Mada, Yogyakarta, Indonesia; 2 Department of Surgery, Dr. Sardjito General Hospital, Faculty of Medicine, Public Health and Nursing, Universitas Gadjah Mada, Yogyakarta, Indonesia; 3 Neurology Department, Dr. Sardjito General Hospital, Faculty of Medicine, Public Health and Nursing, Universitas Gadjah Mada, Yogyakarta, Indonesia; 4 Anatomical Pathology Department, Dr. Sardjito General Hospital, Faculty of Medicine, Public Health and Nursing, Universitas Gadjah Mada, Yogyakarta, Indonesia

**Keywords:** spinal cord injury, balloon compression, animal model, canine, translational study

## Abstract

Spinal cord injury (SCI) is a life-altering condition that severely impacts an individual’s functional capabilities and has significant implications for both the individual and society. Large animal models are crucial for understanding the pathology and biomechanics of SCI. Dogs (*Canis lupus familiaris*) are promising models for SCI research due to their anatomical and histopathological similarities to humans. Balloon compression is an established method for inducing controlled SCI in canines. In this study, we optimized a balloon compression procedure for inducing SCI in dogs, aiming to develop a reliable model for future in vivo studies. Our methodology successfully induced total motoric loss in canines, observed for seven days, a critical period for therapeutic interventions. Histopathological examinations using Luxol fast blue (LFB) staining revealed total demyelination in intralesional samples, confirming the structural damage caused by balloon compression. We concluded that a balloon compression model at the T10-T11 vertebral level, with an inflated balloon volume of 1.0 ml, induced SCI while minimizing the risk of balloon rupture. Longer duration of compression ensures total paralysis in this model, providing a platform for testing therapeutic interventions during the acute phase of SCI. The canine model generated consistent data and facilitated straightforward observational findings.

## INTRODUCTION

Spinal cord injury (SCI) is a life-altering condition that significantly impacts an individual’s functional capabilities, including the ability to perform simple daily tasks, sensory perception, motor function, and, in some instances, autonomic functions. This, in turn, poses a significant economic and functional burden both on an individual and societal level. Over the years, considerable efforts have been made to develop effective treatments to improve neurological function in patients with SCI. Despite these efforts, many research studies and trials show poor prognoses [1,2]. Consequently, there has been a shift towards trying to understand the fundamental biology and biomechanics of SCI.

Intervertebral disc diseases, including disc extrusions and herniated discs, are approximately 5 to 20 cases per 1,000 persons annually, with a prevalence reaching 1–3% of patients [3]. These conditions are most common between the ages of 30 and 50, with a male-to-female ratio of 2:1. Aging is the primary factor related to disc extrusions at any vertebral level, as it leads to the degeneration of intervertebral discs due to the loss of proteoglycans. This results in decreased osmotic pressure within the disc matrix and subsequent loss of water molecules, which ultimately alters the mechanical properties of the disc [4]. Other factors that contribute to this disease include obesity, which predominantly affects the lumbar region, smoking, and occupations with poor ergonomic conditions [4]. Smoking further increases damage by limiting the transportation of nutrients into the intervertebral disc [5]. Obesity and poor ergonomics cause disc damage through mechanical stress [4,6]. Clinical conditions may vary depending on the severity of the disc disease, ranging from complaints of lower back pain or a burning sensation to numbness and tingling, and severe cases may result in motor weakness and disability [6].

Animal models are important in improving our understanding and knowledge of SCI pathology. These models allow the analysis of biological and anatomical events under a controlled setting [7]. Thus, for an animal injury model to be successful, it must share similarities with humans in terms of causation and mechanism of injury, physiology, and pathology of events throughout the injury. Small animal models, such as guinea pigs and rodents, are commonly used for SCI studies [7]. These animal models have the advantage of low cost of use, ease of handling, high availability, and a good understanding of the anatomy. Furthermore, rodent models have been shown to have similarities to human pathology and share genomic patterns similar to those of humans [7]. Although much has been learned about SCI pathology through small animal models, these models are best suited for preliminary studies [7]. Discrepancies in body and spine sizes compared to humans may cause inconsistent results when applied directly in a clinical setting. This has often been demonstrated in studies showing effectiveness in treating small animals, which do not translate to larger animals [8]. Moreover, the application of current human SCI management techniques to these small animals is limited due to their size differences.

The primary approach to clinical SCI management involves mechanical decompression and stabilization of the spine. Larger animal models offer significant advantages, as therapies can be more easily applied than smaller models. Therefore, large animal models are needed to complement the advancements made on small animal models.

Several studies have shown the potential of dogs as SCI models. Canines have a vertebral structure similar to humans, consisting of two vertebrae with a disc-shaped soft tissue in between, functioning as a segment. Both humans and dogs have a similar cause of spinal cord injury, which is intervertebral disc disease. Canines may also experience dorsal pain because of intervertebral disc degeneration, which may subsequently result in herniation similar to humans. This is a major contributor to neurological problems in canines and a leading cause of euthanasia in young dogs (under 10 years old). The pathological changes observed in the spinal cord after SCI are very similar between dogs and humans [9]. This close resemblance makes canines a valuable large animal model for studying SCI and developing new treatments [9].

While canines are valuable models for SCI research, their diverse body sizes across breeds require careful consideration during study design. Previous studies addressed this by using standardized weight categories, creating more uniform groups, and enhancing the findings' relevance to humans [10]. Research has already demonstrated the potential of large animal models like dogs and goats for SCI studies [7]. The use of large animal models offers several advantages. Their larger size allows for more intricate manipulations and larger instruments, facilitating the translation from animal studies to human applications.

Inducing spinal cord injury in animal models poses significant challenges. Dogs are the most common large animals used for SCI models, and injury mechanisms often involve contusion, where a transient force is applied to mimic sudden damage and displacement of the spinal cord [3]. Ideally, the procedure should replicate mechanisms found in human SCI and be consistent enough to yield reproducible results [1]. Past methods included controlled transection, contusion, weight dropping, and ballooning compression, generating a different SCI response [1]. Since intervertebral disc extrusion (IVDE), the most common cause of SCI in humans, induces SCI by reducing the area that the spinal cord can occupy in the spinal canal, the balloon compression procedure, which follows a similar mechanism may offer the best potential for use in SCI studies. First developed by Tarlov *et al*. [11], this procedure involves using a balloon catheter to compress the spinal cord in dogs, allowing researchers to study the effects of different compression durations on recovery. The authors concluded that the functional recovery after SCI depended on the magnitude and duration of force on the spinal cord. However, variable results, such as medium-sized balloon compression for 36 hours, showed complete recovery within four days. A study by Lee *et al*. [12] further developed the balloon compression method by measuring spinal canal occlusion using transverse CT imaging, with balloon volumes ranging between 50 µl and 150 µl, producing 50-75% spinal canal occlusion. Fukuda *et al*. [13] successfully developed a balloon compression model with volumes of 1.5 ml for 10 minutes, causing irreversible paraplegia in dogs [13]. However, Tarlov *et al*. initially reported difficulty maintaining large balloon volume sizes due to consistent balloon deflation caused by the midline vertebral ridge [11]. Lim *et al*. [14] showed consistent SCI in all subjects with up to 1.0 ml balloon compression, which conflicted with the results of Fukuda *et al*. [13]. This could be due to larger canal sizes at the L1-L2 level. A higher spinal location in the canine model may compensate for smaller balloon volumes, reducing rupture risk. Additionally, increasing compression duration can enhance the likelihood of SCI induction. As CT scan availability may be a factor in some research facilities, using simple X-ray scanning to confirm SCI induction may be a suitable alternative.

The balloon–induced method shows promise as a simple technique for SCI animal models, reducing collateral damage to surrounding tissue [7]. This method offers the consistency and reproducibility needed for SCI studies in animal models. It involves minimal exposure to the surgical site, reducing infection risks. However, balloon size and compression duration inconsistencies highlight the need for further development.

This study aimed to develop a balloon compression model in canines at a higher vertebrae level (T10-T11), using a 1.0 ml inflated balloon for 6 hours. Developing a standard reproducible large animal model for SCI is vital in bridging the gap between experimental therapies and clinical applications. This study hopes to advance the understanding of SCI pathology and neurological manifestations, given the significant impact of SCI on daily activities, neurological functions, and emotions.

## MATERIAL AND METHODS

### Study design

This study aimed to develop an optimal balloon compression-induced SCI model using dogs (*Canis lupus familiaris*) as subjects to represent large mammalian models. The study was conducted at the Prof. Soeparwi Animal Hospital of Gadjah Mada University between May 2022 and September 2023.

### Study animals

Consecutive sampling was employed to select mongrel (mixed breed) dogs for the animal model. Mongrels were preferred due to their spinal and vertebral structures resembling human anatomy. Animals were obtained from the local stray dog shelter. Male dogs, aged 1–5 years, healthy, and weighing 10–15 kilograms, were chosen to ensure size homogeneity. Weight was the primary factor used to determine animal size consistency. Dogs with neurological disorders (cold or heat sensation test > 10 seconds), musculoskeletal disorders (canine Basso–Beattie–Bresnahan [cBBB] score <17), active infections, or other pathological conditions were excluded from the study.

### Pre-surgical preparation

**Figure 1:**
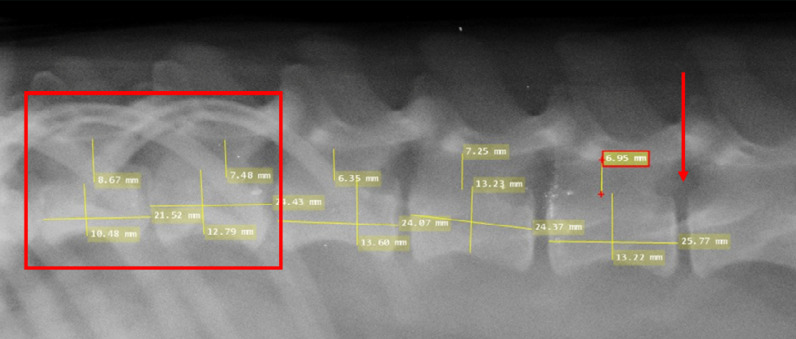
The vertebrae were measured under X-ray imaging, confirming spinal canal diameters <10 mm. The red box indicates the site of the compression target at the T10-T11 vertebrae level. The red arrow indicates the site for balloon insertion below the L1 vertebrae.

Before surgery, all dogs underwent a comprehensive health screening conducted by qualified veterinarians. This screening aimed to establish baseline health data and ensure animal welfare throughout the study. This included obtaining hematological profiles, liver function, and kidney function tests to screen for any abnormalities that could interfere with the study. Prophylactic anti-helminthic drugs and antibiotics were administered to prevent parasitic infestation or bacterial infection that may occur undetected before surgery. Specifically, the dogs received amoxicillin 10 mg/kg and a combination of praziquantel 50mg, pyrantel embonate 144 mg, and febantel 150 mg as anti-helminthic treatment for one day. The site of the incision was marked under radiologic X-ray guidance. The diameter of the spinal canal was measured under radiologic X-ray guidance in the anteroposterior (AP) and lateral positions. Dogs with a spinal canal diameter above 10 mm ± 0.5 mm at the T10-T11 level were excluded due to the possibility of incomplete SCI induction. The target occlusion of the spinal canal was 100%, with a 5% margin of error (Figure 1). The lumbar vertebrae were marked at the L1 level for the initial incision for all dogs. To reach the T10-T11 compression target, the balloon was inserted approximately 5 cm from L1, based on radiologic measurements.

### Surgical procedure

To induce SCI, balloon compression was employed to mimic compression-stimulated SCI, a common mechanism in humans. The dogs were anesthetized using ketamine 10 mg/kg intravenously, and sedation was maintained throughout the procedure using sevoflurane 1–2% by inhalation route. An endotracheal tube was inserted to maintain respiratory function using a ventilator, and a urinary catheter was placed to assist with urination during the procedure and monitor post-surgery urinary function.

**Figure 2:**
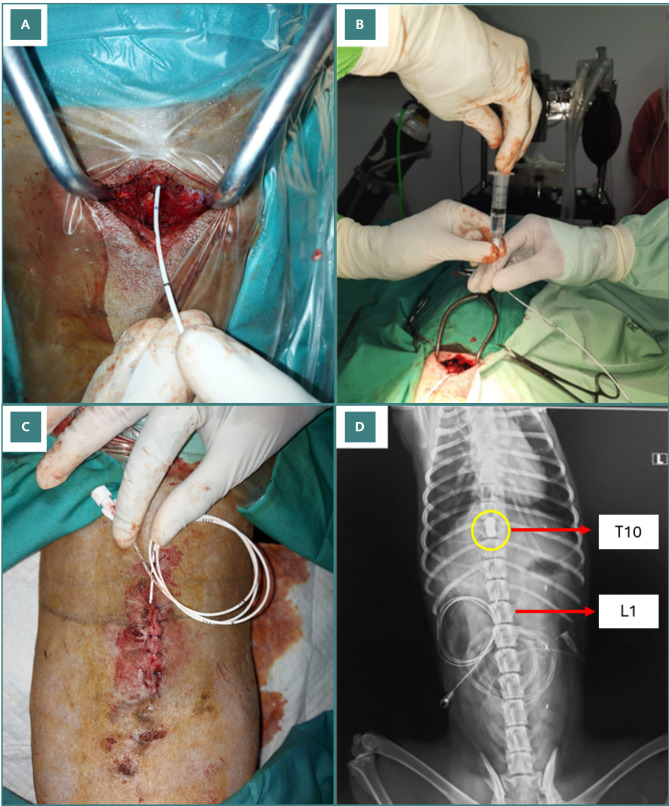
. Induction surgery. A, Fogarty catheter (FC) insertion through the opening of the perforated lamina into the epidural space. B, Balloon inflated with 1 mL of iohexol contrast agent until the epidural space was filled. C, The surgical site was sutured, the catheter was sealed to prevent leakage, and the balloon was left inflated. D, Six hours after the procedure, X-ray imaging was repeatedly performed to confirm whether the balloon was still inflated. The yellow circle shows the location of the inflated balloon catheter. Red labels indicate relevant vertebral levels.

A 5-cm incision was made along the midline of the dog's back, starting at the level of the L1 vertebra. The surgeon dissected through the layers of skin, subcutaneous tissue, and muscle. The underlying bone was then exposed, and the vertebral lamina of L1 was identified, providing the surgical access point for the next stage of the procedure. Decompression was done using a 1 mm curve curette to release the flavum ligament. A laminotomy procedure was then done in the lower lamina and upper lamina of the targeted interlaminar space using a 1 mm and 2 mm Kerrison rongeur to clear the area for the Fogarty catheter (FC) entrance. The FC was then inserted through the opening of the perforated lamina and placed in the epidural space (Figure 2A). From there, the balloon was inserted cranially approximately 5 cm to the level of the T10–T11 segment of the spinal cord. At this level, the balloon was inflated with 1.0 mL iohexol contrast agent until the epidural space was filled (Figure 2B), ensuring no air was present within the inflated balloon. An involuntary jerk reaction was observed at this stage and was used to indicate induced paralysis. After the balloon insertion, the surgical site was closed by suturing (Figure 2C).

The catheter was sealed to prevent leakage, and the balloon was left inflated for six hours post-surgery. Post-operative X-ray examinations were carried out to confirm the proper position of the balloon at the T10–T11 level, as well as the complete occlusion to reach 100% of the spinal canal. Six hours after the procedure, X-ray imaging was repeated to verify the condition of the balloon, ensuring it remained inflated (Figure 2D). Upon confirmation, the balloon was gently deflated and removed from the epidural space without disrupting the previous surgical suture.

### Post-surgical evaluation

After surgery, the dogs were provided with wheelchairs to aid in daily movement and stability. Constant monitoring and care were carried out by qualified veterinarians until the end of the study. All necessary drugs and assistive devices were prepared. Amoxicillin 10 mg/kg and metronidazole 15 mg/kg were administered to the dogs for five days to prevent any infections that may occur post-surgery. Additionally, Sulpidon (2 mg/kg) was given as needed to manage any signs of pain.

Observations were made one day before surgery, six hours post-surgery, three days post-surgery, and seven days post-surgery to represent the acute phase of SCI. Daily activity, movement, and neurological function were routinely assessed, and any notable findings were reported.

### Neurological evaluation

Neurological assessments were conducted to evaluate the impact of SCI on motor function, sensory perception, and deep tendon reflexes in the dogs. Motor function was evaluated using the canine Basso–Beattie–Bresnahan (cBBB) score, as detailed in Table 1 [7]. Voluntary tail wag was also observed as an indicator of motor function. Adapted from a study by Gorney *et al*. [8], sensory function was assessed by observing withdrawal responses of the limb, sudden motion, growling, or head turning in response to harmful stimuli (heat at 49°C and cold at 5°C). The response time was recorded, with maximum times of 30 seconds for heat and 60 seconds for cold stimuli. If the dogs did not exhibit any withdrawal response, the maximum time was reported. Tests were conducted three times on both lower limbs, and the most apparent response was reported. Patellar reflexes were assessed to determine whether motor or sensory function changes were due to successful injury induction or acute spinal shock. An absence of reflex responses would indicate spinal shock. Reflex responses were graded as follows: 1 = hyporeflexia, 2 = normoreflexia, and 3 = hyperreflexia.

**Table 1:** Canine Basso–Beattie–Bresnahan Criteria [7]

0 = No observable hindlimb (HL) movement
1 = Slight movement of one or two joints
2 = Extensive movement of one joint, or extensive movement of one joint and slight movement of one other joint
3 = Extensive movement of two joints
4 = Slight movement of all three joints of the HL
5 = Slight movement of two joints and extensive movement of the third
6 = Extensive movement of two joints and slight movement of the third
7 = Extensive movement of all three joints in the HL
8 = Plantar placement of the paw with no weight support
9 = Plantar placement of the paw with weight support only when stationary, or occasional, frequent, or consistent weight-supported dorsal stepping and no plantar stepping
10 = Occasional weight-supported plantar steps; no FL–HL coordination
11 = Frequent to consistent weight-supported plantar steps and no FL–HL coordination
12 = Frequent to consistent weight-supported plantar steps and occasional FL–HL coordination
13 = Frequent to consistent weight-supported plantar steps and frequent FL–HL coordination
14 = Consistent weight-supported plantar steps, consistent FL–HL coordination, and predominant paw position is externally rotated when it makes initial contact as well as just before it is lifted off; or frequent plantar stepping, consistent FL–HL coordination, and occasional dorsal stepping
15 = Consistent plantar stepping and consistent FL–HL coordination and no toe clearance or occasional toe clearance; predominant paw position is parallel to the body or internally rotated at initial contact
16 = Consistent plantar stepping and consistent FL–HL coordination and toe clearance occur frequently; predominant paw position is parallel or internally rotated at initial contact and externally rotated at liftoff
17 = Consistent plantar stepping and consistent FL–HL coordination and toe clearance occur frequently; predominant paw position is parallel or internal at initial contact and liftoff
18 = Consistent plantar stepping and consistent FL–HL coordination and toe clearance occur consistently; predominant paw position is parallel or internal at initial contact and liftoff. Trunk instability is present
19 = Consistent plantar stepping and consistent FL–HL coordination and toe clearance occur consistently during forward limb advancement; predominant paw position is parallel or internal at initial contact and liftoff. Trunk instability is not observed

### Daily activity

Daily functions such as physical activity, movement, urination, defecation, and appetite were monitored during the observational period. Physical activity was recorded on video for four minutes after the dogs were released from their cages. Activity levels were categorized as hyperactive, normoactive, hypoactive, or absent.

Urination was observed as either spontaneous or catheter-assisted, and defecation was noted as spontaneous or manually assisted. Appetite was assessed based on the dogs' willingness to eat food provided by the researcher. Appetite levels were categorized as normal, increased, decreased, or absent. Normal appetite was defined as consuming 2–5% of body weight per day, increased appetite as more than 5%, decreased appetite as less than 2%, and no appetite as no food intake within a day.

### Histopathological investigation

Following the observation period, the animals were euthanized on the 10th day post-induction using an injection of propofol (6 mg/kg) followed by MgSO4 (20 mg). The spinal cord specimens were subsequently obtained through postmortem surgical extractions. Samples were preserved in formalin and stained with hematoxylin and eosin (H&E) and Luxol fast blue (LFB) to observe hemorrhage, necrosis, inflammation, and structural damage through demyelination. Observations were made in three areas: the injury site, below the injury site, and above the injury site, with distances determined by previous X-ray imaging. The length of the corpus vertebrae determines the length between the injury site and the superior and inferior sites. Evaluations were done by anatomical pathologist specialists.

Structural damage was observed qualitatively through the classification shown in Table 2 [15]. Inflammation and hemorrhage were evaluated semi-quantitatively using the classifications in Tables 3 and 4 [16,17]. Necrosis was described qualitatively as either observed (+) or not observed (-) [16,17].

**Table 2:** Structural damage assessment in LFB staining

Description	Score
Normally myelinated area	1
Partially demyelinated area (>1/2 cross-sectional intramedullary area)	2
Completely demyelinated area	3

**Table 3:** Hemorrhage score

Description	Score
No hemorrhage	0
Scattered ring hemorrhage	1
Coalescing ring hemorrhage and diffuse spread of erythrocytes in the parenchyma	2
Massive hemorrhage	3

**Table 4:** Inflammation score

Description	Score
No inflammation	0
Cellular infiltration only in the perivascular areas and meninges	1
Mild cellular infiltration (<1/3 of total white matter)	2
Moderate cellular infiltration (>1/3 of total white matter)	3
Infiltration of inflammatory cells observed in the whole white matter	4

## RESULTS

The balloon compression procedure for inducing SCI was successfully performed on ten dogs. The position of the balloons was confirmed by X-ray imaging post-surgery and again six hours later. For ease of observation, the dogs were numbered from one to ten. Pre-surgical blood and urine analyses showed no abnormalities in any of the dogs. None of the dogs were observed to have any active infections, neurological or musculoskeletal disorders, or any observable pathological disorder.

### Neurological evaluation

**Figure 3:**
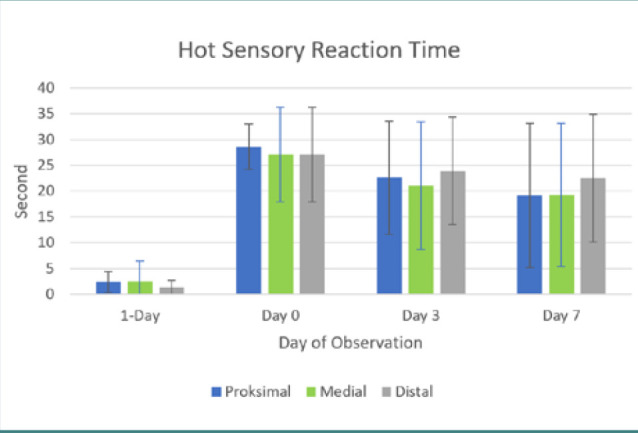
Chart of hot sensory reaction time

**Figure 4:**
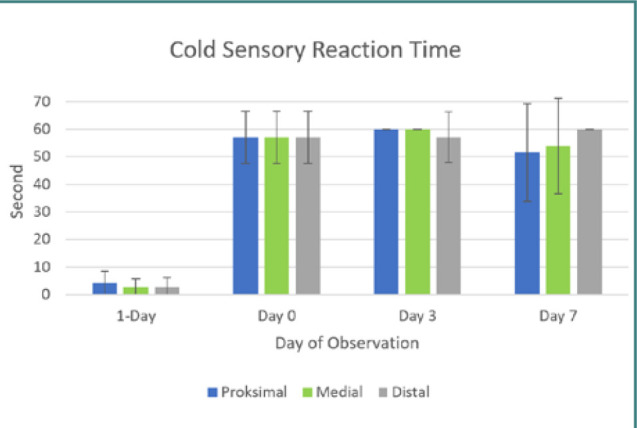
. Chart of cold sensory reaction time

Motor function in both posterior limbs, assessed using the cBBB scoring system, completely ceased post-balloon compression surgery in all ten dogs with a cBBB score of 0 maintained throughout the seven-day observation period. All dogs had an instant loss in voluntary tail wag directly after the balloon compression procedure. Sensory function significantly decreased in all dogs except for dog five. Sensory function remained compromised in all dogs, but dogs 1, 6, and 7 showed increased hot sensory responses by day three post-SCI induction. Dogs 1, 2, 6, 7, 9, and 10 showed increased reflex response directly post-surgery, whereas dogs 3 and 8 showed delayed hyper-reflex only appearing three days post-surgery (Table 5 and Figures 3 and 4).

**Table 5:** Neurological assessment of deep tendon reflex and sensory reaction times (1 = hyporeflexia; 2 = normoreflexia; and 3 = hyperreflexia).

			1-Day Before Surgery	0-Day After Surgery	3-Days After Surgery	7-Days After Surgery
**Dog 1**	Motor function		19	0	0	0
	Deep tendon reflex	Left	2	3	3	3
		Right	2	3	3	3
	Hot sensory reaction time (Second)	Proximal	<1	30	6	3
		Medial	<1	30	12	6
		Distal	<1	30	30	30
	Cold sensory reaction time (Second)	Proximal	<1	60	60	17
		Medial	<1	60	60	60
		Distal	<1	60	60	60
**Dog 2**	Motor function		19	0	0	0
	Deep tendon reflex	Left	2	3	3	3
		Right	2	3	3	3
	Hot sensory reaction time (Second)	Proximal	<1	30	30	30
		Medial	<1	30	30	30
		Distal	<1	30	30	30
	Cold sensory reaction time (Second)	Proximal	<1	60	60	60
		Medial	<1	60	60	60
		Distal	<1	60	60	60
**Dog 3**	Motor function		19	0	0	0
	Deep tendon reflex	Left	2	2	2	3
		Right	2	2	2	3
	Hot sensory reaction time (Second)	Proximal	1	30	30	30
		Medial	1	30	30	30
		Distal	1	30	30	30
	Cold sensory reaction time (Second)	Proximal	5	60	60	60
		Medial	1	60	60	60
		Distal	2	60	60	60
**Dog 4**	Motor function		19	0	0	0
	Deep tendon reflex	Left	2	2	2	2
		Right	2	2	2	2
	Hot sensory reaction time (Second)	Proximal	5	30	30	30
		Medial	1	30	30	30
		Distal	1	30	30	30
	Cold sensory reaction time (Second)	Proximal	1	60	60	60
		Medial	2	60	60	60
		Distal	1	60	60	60
**Dog 5**	Motor function		19	0	0	0
	Deep tendon reflex	Left	2	2	2	2
		Right	2	2	2	2
	Hot sensory reaction time (Second)	Proximal	1	16	1	4
		Medial	1	1	1	2
		Distal	1	1	4	12
	Cold sensory reaction time (Second)	Proximal	7	30	60	19
		Medial	7	30	60	5
		Distal	10	30	31	60
**Dog 6**	Motor function		19	0	0	0
	Deep tendon reflex	Left	2	3	3	3
		Right	2	3	3	3
	Hot sensory reaction time (Second)	Proximal	2	30	21	1
		Medial	1	30	17	2
		Distal	1	30	18	1
	Cold sensory reaction time (Second)	Proximal	1	60	60	60
		Medial	1	60	60	54
		Distal	1	60	60	60
**Dog 7**	Motor function		19	0	0	0
	Deep tendon reflex	Left	2	3	3	3
		Right	2	3	3	3
	Hot sensory reaction time (Second)	Proximal	5	30	18	4
		Medial	1	30	1	3
		Distal	1	30	7	2
	Cold sensory reaction time (Second)	Proximal	13	60	60	60
		Medial	2	60	60	60
		Distal	2	60	60	60
**Dog 8**	Motor function		19	0	0	0
	Deep tendon reflex	Left	2	2	3	2
		Right	2	2	3	2
	Hot sensory reaction time (Second)	Proximal	5	30	30	30
		Medial	7	30	30	30
		Distal	5	30	30	30
	Cold sensory reaction time (Second)	Proximal	6	60	60	60
		Medial	5	60	60	60
		Distal	4	60	60	60
**Dog 9**	Motor function		19	0	0	0
	Deep tendon reflex	Left	2	3	3	3
		Right	2	3	3	3
	Hot sensory reaction time (Second)	Proximal	2	30	30	30
		Medial	12	30	30	30
		Distal	2	30	30	30
	Cold sensory reaction time (Second)	Proximal	8	60	60	60
		Medial	8	60	60	60
		Distal	7	60	60	60
**Dog 10**	Motor function		19	0	0	0
	Deep tendon reflex	Left	2	3	3	3
		Right	2	3	3	3
	Hot sensory reaction time (Second)	Proximal	3	30	30	30
		Medial	1	30	30	30
		Distal	1	30	30	30
	Cold sensory reaction time (Second)	Proximal	1	60	60	60
		Medial	2	60	60	60
		Distal	1	60	60	60

### Post-surgical daily activity

Normal urination and defecation were observed in all dogs throughout the observation period. Urination was assisted by catheterization in all dogs throughout the observation period. Following the balloon compression procedure, dogs 1, 2, 4, 5, and 10 displayed a decreased appetite, while dogs 7 and 8 showed a notable increase in appetite. Dogs 3 and 9 maintained their normal eating habits. Dogs 7 and 8 showed hyperactive behavior post-compression procedure, which was remarkable. Dogs 3, 4, 5, 6, 9, and 10 had passive behaviors post-compression procedure. Dogs 1 and 2 maintained their usual activity levels compared to pre-procedure observations (Table 6).

**Table 6:** Daily activity levels

		1-Day Before Surgery	0-Day After Surgery	3-Day After Surgery	7-Day After Surgery
**Dog 1**	Appetite	Normal	Decreased	Decreased	Decreased
	Urination	Spontaneous	Catheter-assisted	Catheter-assisted	Catheter-assisted
	Defecation	Spontaneous	Spontaneous	Spontaneous	Spontaneous
	Physical Activity	Normoactive	Normoactive	Normoactive	Normoactive
**Dog 2**	Appetite	Normal	Normal	Decreased	Decreased
	Urination	Spontaneous	Catheter-assisted	Catheter-assisted	Catheter-assisted
	Defecation	Spontaneous	Spontaneous	Spontaneous	Spontaneous
	Physical Activity	Normoactive	Normoactive	Normoactive	Normoactive
**Dog 3**	Appetite	Normal	Normal	Normal	Normal
	Urination	Spontaneous	Catheter-assisted	Catheter-assisted	Spontaneous
	Defecation	Spontaneous	Spontaneous	Spontaneous	Spontaneous
	Physical Activity	Normoactive	Passive	Passive	Normoactive
**Dog 4**	Appetite	Normal	Decreased	Decreased	Decreased
	Urination	Spontaneous	Catheter-assisted	Catheter-assisted	Catheter-assisted
	Defecation	Spontaneous	Spontaneous	Spontaneous	Spontaneous
	Physical Activity	Normoactive	Passive	Passive	Passive
**Dog 5**	Appetite	Normal	Decreased	Decreased	Decreased
	Urination	Spontaneous	Catheter-assisted	Catheter-assisted	Catheter-assisted
	Defecation	Spontaneous	Spontaneous	Spontaneous	Spontaneous
	Physical Activity	Normoactive	Passive	Normoactive	Normoactive
**Dog 6**	Appetite	Normal	Decreased	Normal	Decreased
	Urination	Spontaneous	Catheter-assisted	Catheter-assisted	Catheter-assisted
	Defecation	Spontaneous	Spontaneous	Spontaneous	Spontaneous
	Physical Activity	Normoactive	Passive	Passive	Passive
**Dog 7**	Appetite	Normal	Normal	Increase	Increase
	Urination	Spontaneous	Catheter-assisted	Catheter-assisted	Catheter-assisted
	Defecation	Spontaneous	Spontaneous	Spontaneous	Spontaneous
	Physical Activity	Normoactive	Passive	Hyperactive	Hyperactive
**Dog 8**	Appetite	Normal	Increase	Increase	Increase
	Urination	Spontaneous	Catheter-assisted	Catheter-assisted	Catheter-assisted
	Defecation	Spontaneous	Spontaneous	Spontaneous	Spontaneous
	Physical Activity	Normoactive	Hyperactive	Hyperactive	Hyperactive
**Dog 9**	Appetite	Normal	Normal	Normal	Normal
	Urination	Spontaneous	Catheter-assisted	Catheter-assisted	Catheter-assisted
	Defecation	Spontaneous	Spontaneous	Spontaneous	Spontaneous
	Physical Activity	Normoactive	Passive	Passive	Normoactive
**Dog 10**	Appetite	Normal	Decreased	Decreased	Decreased
	Urination	Spontaneous	Catheter-assisted	Catheter-assisted	Catheter-assisted
	Defecation	Spontaneous	Spontaneous	Spontaneous	Spontaneous
	Physical Activity	Normoactive	Passive	Passive	Passive

### Other notable findings

Under paraplegic conditions, some notable additional findings were observed. These findings were found on the third-day post-surgery in dogs 6 and 10. In dog 6, bloody urine was reported, whereas in dog 10, edema and rash on the testis were found on day 3 post-compression procedure. These rashes were persistent until the study ended. On the fifth day, the edema in dog 10 expanded to the inguinal region and hindlimb. The oedema ceased on the seventh day. On the same day, dog 10 began displaying unpredictable and irrational behavior, biting its genitals, which led to hemorrhaging and subsequent necrosis by the tenth day despite veterinary treatment. Frictional injury from the jagged cage floor may have contributed to this injury, as the dog could only haul its hindlimbs and lower body.

### Histopathological investigation

**Figure 5:**
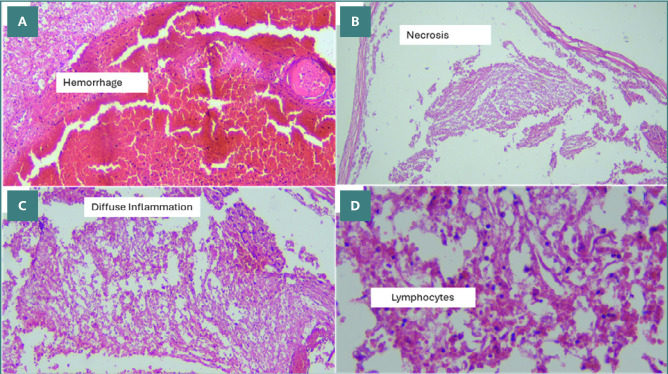
H&E findings at the intralesion level in dog 2. A, 100x magnification: Massive intramedullary hemorrhage (score 3), showing the highest scoring for hemorrhage. B, 100x magnification: Presence of subdural necrosis. C, 100x magnification: Intramedullary diffuse inflammation (score 4), showing the highest inflammation score. D, 400x magnification: Intramedullary visualization of lymphocyte infiltration.

Using H&E staining, we observed moderate cellular inflammation in all intralesional samples, with a median score of 3 (Figure 5). The inflammation scores ranged from a minimum of two to a maximum of four. The hemorrhage observed in intralesional samples showed a median score of two, with a minimum and maximum score of one and three, respectively. Necrosis was observed in the intralesional samples of all animals except for dog number seven. The complete distribution of findings from the H&E staining can be seen in Table 7.

**Table 7:** Histopathological findings (H&E)

Subject	Location	Inflammation score	Hemorrhage score	Necrosis
**Dog 1**	Supra-lesion	1	0	(-)
	Intra-lesion	3	2	(+)
	Infra-lesion	1	0	(-)
**Dog 2**	Supra-lesion	1	0	(-)
	Intra-lesion	4	3	(+)
	Infra-lesion	1	0	(-)
**Dog 3**	Supra-lesion	1	1	(-)
	Intra-lesion	3	3	(+)
	Infra-lesion	0	0	(-)
**Dog 4**	Supra-lesion	1	2	(+)
	Intra-lesion	2	2	(+)
	Infra-lesion	1	1	(-)
**Dog 5**	Supra-lesion	1	2	(-)
	Intra-lesion	3	2	(+)
	Infra-lesion	1	1	(-)
**Dog 6**	Supra-lesion	1	1	(-)
	Intra-lesion	4	1	(+)
	Infra-lesion	1	2	(+)
**Dog 7**	Supra-lesion	1	0	(-)
	Intra-lesion	2	1	(-)
	Infra-lesion	1	2	(+)
**Dog 8**	Supra-lesion	1	1	(-)
	Intra-lesion	2	3	(+)
	Infra-lesion	2	2	(+)
**Dog 9**	Supra-lesion	0	0	(-)
	Intra-lesion	2	1	(+)
	Infra-lesion	1	1	(+)
**Dog 10**	Supra-lesion	0	1	(-)
	Intra-lesion	3	1	(+)
	Infra-lesion	2	3	(-)

**Figure 6:**
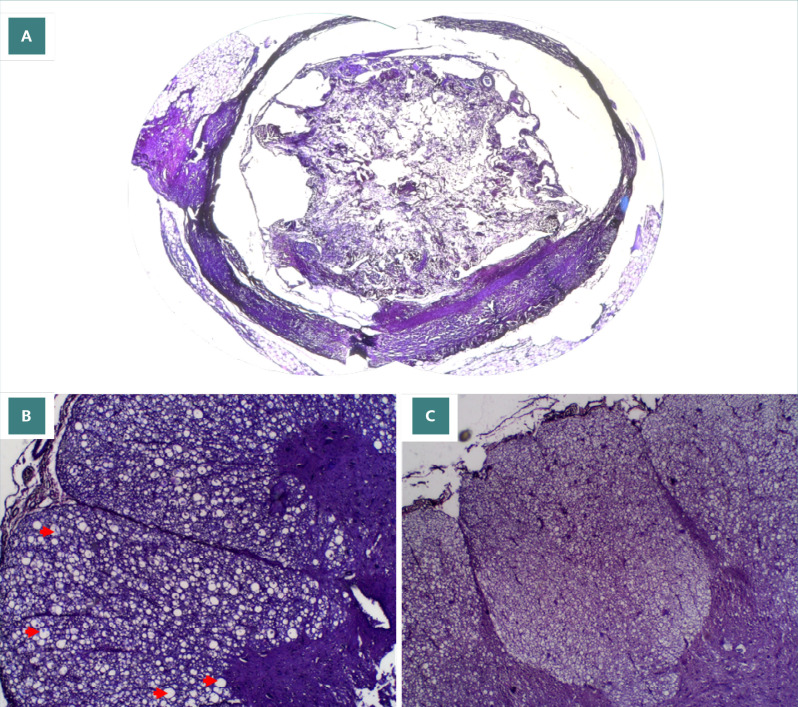
LFB findings for normal myelination, vacuole formation, and total demyelination. A, 40x magnification: Total demyelination of intralesion sample in dog 3. B, 100x magnification: Vacuole formation of lateral cross section in supra lesional sample of dog 1. C, 100x magnification: Posterior section of infralesional sample of dog 10 showing normal myelination

Luxol fast blue (LFB) staining revealed complete demyelination in the injured spinal cord segments (intralesional) of all dogs except dog 4. Interestingly, dog 4 exhibited complete demyelination in the area above the injury site (supralesional) (Figure 6 A-C). All other spinal cord segments, both above (supralesional) and below (infralesional) the injury site in all dogs, displayed normal myelination. Vacuolar formations were observed in multiple samples, indicating the start of apoptosis of regional cells. The complete distribution of findings from the LFB staining can be seen in Table 8.

**Table 8:** LFB staining findings

Subject	Location	Score	Additional Findings
**Dog 1**	Supra Lesion	1	Vacuolar formation
	Intra Lesion	3	
	Infra Lesion	1	
**Dog 2**	Supra Lesion	1	Vacuolar formation
	Intra Lesion	3	
	Infra Lesion	1	
**Dog 3**	Supra Lesion	1	Vacuolar formation
	Intra Lesion	3	
	Infra Lesion	1	
**Dog 4**	Supra Lesion	3	
	Intra Lesion	2	Vacuolar formation
	Infra Lesion	1	Vacuolar formation
**Dog 5**	Supra Lesion	1	Vacuolar formation
	Intra Lesion	3	
	Infra Lesion	1	Vacuolar formation
**Dog 6**	Supra Lesion	1	Vacuolar formation
	Intra Lesion	3	
	Infra Lesion	1	
**Dog 7**	Supra Lesion	1	Vacuolar formation
	Intra Lesion	3	
	Infra Lesion	1	
**Dog 8**	Supra Lesion	1	Vacuolar formation
	Intra Lesion	3	
	Infra Lesion	1	
**Dog 9**	Supra Lesion	1	Vacuolar formation
	Intra Lesion	3	
	Infra Lesion	1	Vacuolar formation
**Dog 10**	Supra Lesion	1	
	Intra Lesion	3	
	Infra Lesion	1	Vacuolar formation

## DISCUSSION

The spinal cord, a crucial component of the central nervous system (CNS), is the primary pathway for transmitting information between the peripheral nervous system (PNS) and the brain [18,19]. When an injury occurs to the spinal cord, this communication is disrupted. This disruption leads to the paralysis of the limbs innervated by spinal ganglia below the injury level [19]. The disruption extends beyond motor function, potentially compromising sensory and autonomic functions depending on the specific location and severity of the injury. This concept is especially true in mammalian species, such as humans and dogs.

The diameter of the human spinal canal varies across different vertebral regions. It increases from C2 to C5, then decreases at the second thoracic level, goes up to the seventh thoracic level, and remains stable until the 11^th^ thoracic vertebrae level. At the 12^th^ thoracic vertebrae, the spinal canal diameter increases again and continues into the lumbar vertebrae [20]. Canines share similar characteristics, with the largest spinal canal diameter at the atlas, with a diameter of approximately 1 cm. This tapers down the spinal canal, reaching roughly half the diameter by the caudal end [21]. Like humans, canines experience spinal canal enlargement at the sixth and seventh cervical vertebrae, as well as the fourth and fifth lumbar vertebrae [22]. Clinically, this anatomical variation implies that injuries, stenosis, or masses occurring at the thoracic level are more likely to cause complete paralysis in lower segments due to the narrower spinal canal diameter in this region. Although more attention is paid to cervical or lumbar disc herniations, thoracic disc herniations, notably of the lower segments, have been shown to cause progressive paraplegia, as well as bowel and bladder dysfunctions [23]. Similar IVDD issues have been reported in dogs, with the protrusion type being a leading cause of acute paralysis [24]. Theoretically, balloon compression at a higher level, such as T10-T11, increases the likelihood of complete SCI while requiring smaller balloon volumes. In canines, acute cases of intervertebral disc diseases may cause severe lesions leading to compressive and contusive injury of the spinal cord. Conversely, slowly progressing IVDD may lead to milder, gradual clinical symptoms [25]. Findings from dogs with acute spinal cord injury following intervertebral disc herniations show similar gross and histopathological characteristics with humans with traumatic myelopathies, although notable anatomical and physiological differences between canine and human nervous tracts are present [26].

In this study, motor and sensory functions were successfully disrupted in all subjects directly after SCI induction through balloon compression. This effect persisted throughout the seven-day experimental period. In terms of sensory function, responses towards heat and cold stimuli decreased following induction surgery in all dogs except dog 5. By the third day post-surgery, dogs 1,6 and 7 showed an increased response toward hot sensory stimuli while maintaining a decreased response to cold stimuli. Tarlov *et al*. [11] found similar results in dogs with thoracolumbar spine injuries, observing a loss of motor function but retention of pain sensation. Laitinen and Puerto investigated the relationship between intact pain perception and the prognosis of dogs with SCI, discovering a correlation between the loss of pain sensation and increased morbidity and mortality rates [27]. In their study, pain sensibility was tested by applying high-pressure stimuli, in their case a pinprick, to the plantar surface of the paw. This study adapted the methodology of Gorney *et al*. [18] by employing thermal stimuli to assess pain perception. Gorney *et al*. also utilized mechanical stimuli and reported that SCI-affected dogs had a higher threshold in mechanical and thermal sensation than uninjured dogs [18]. In contrast to our findings, the dogs in Gorney *et al*.'s study did not experience a spontaneous return of sensory function. This discrepancy might be attributed to an incomplete posterior cord compression (location of sensory nerve pathways), allowing recovery within three days post-surgery. The mechanism is still poorly understood, and no other study has reported similar findings.

In mammals, an involuntary motor response, known as reflex activity, protects the animal when in danger [28]. Reflexes can be divided into physiologic and pathologic reflexes. Physiological reflexes are observed in healthy limbs when a harmful stimulus is introduced, whereas pathological reflexes can only be elicited when CNS function is interrupted [19,27,28]. Normally, an uninterrupted CNS inhibits excessive or otherwise useless reflex movement [19,27,28]. Nonetheless, in patients with SCI or traumatic brain injury (TBI), this inhibitory function ceases to exist, and pathological reflexes resurface [19,29-31]. In the case of spinal shock, symptoms such as paraplegia and bowel and bladder dysfunction can mimic those of successful SCI induction. However, observing reflex functions, which are retained or heightened in SCI, can help differentiate between the two. In this study, the physiological reflexes observed in animals followed this concept. Dogs 1, 2, 6, 7, 9, and 10 had immediate hyperreflexia with increased tendon responses post-surgery. Dogs 3 and 8 showed delayed hyperreflexia, indicating that the loss of inhibitory functions following SCI may not be instantaneous.

Since neurological function is compromised in SCI, this condition directly impacts an individual's daily activity and function. In this study, the daily activity of animals consisted of defecation, urination, physical activity, and appetite. All ten dogs had decreased urinary function post-SCI induction and needed the assistance of a urinary catheter. These findings indicate supra sacral spinal cord injuries observed in dogs due to loss of detrusor muscle activation and relaxation and the loss of bladder fullness sensation [32]. Defecation was the sole function that was not interrupted in dogs. Defecation is a complex process involving the intrinsic and extrinsic plexus of the gut, parasympathetic nervous system, somatic motor neuron, and CNS to remove remaining unabsorbable food in the form of feces [33]. Defecation can somatically be controlled since a somatic motor neuron, the pudendal nerve, innervates one of the multiple layers of the anus, the muscle of the external anal sphincter. This nerve controls the opening of the external anal sphincter. The pudendal nerve arises from the S2–S4 level [33-35]. Theoretically, any injury at a specific level of the spinal cord terminates the function of the nerve below the injury site. This implies that the function of the pudendal nerve, which controls defecation and urinary function, should have ceased to function as its level is below the injury site. As the pudendal nerve function is disrupted, feces accumulate inside the gut. However, the accumulation of feces elevates intra-colonic pressure and would later push the feces towards the anus for spontaneous defecation to occur regardless. This may explain why SCI did not alter the defecation function. However, this observation can also be due to the preservation of pelvic and pudendal nerves, which occur in supra-sacral lesions, aligning with our micturition function findings.

Observations of physical activity levels differed greatly among subjects. In dogs 1 and 2, activities varied post-SCI induction compared to pre-SCI induction. Most subjects showed a decrease in physical activity levels, as expected. However, dogs 7 and 8 unexpectedly became more active after the surgery. Carnivorous animals such as dogs have specialized limbs for hunting prey. Unlike herbivores, speed is not a necessary component to survive in nature. The forelimb is designed to carry up to 60% of body weight, while the hindlimb is specialized for speed. Carnivores depend more on forelimb function as carnivores use their forelimbs to lock down prey with their weight [36]. It may explain why a dog can adapt to the paralysis of their hindlimbs, as seen in dogs 7 and 8.

On the seventh day of observation, dog 10 exhibited abnormal behavior by biting its genital area, resulting in bleeding. The cause of this behavior is unclear but may be due to pain, stress from the induction procedure, or an undetected infection. Irritation from the urinary catheter could also be a factor. Initial efforts to measure pain post-induction were made, but due to the subjective nature of these tests, sensory stimulus via heat was used to indicate pain reception below the lesion, as per Gorney *et al*. [18]. However, the behavior in dog 10 suggests the possibility of generalized systemic pain, undetectable through hind limb heat sensory tests, highlighting the need for a standardized visual scoring system for pain in canine models.

In humans, SCI can be divided into acute, subacute, and chronic phases. The acute phase represents the direct initial mechanical injury from the first hour of injury up to seven days. The subacute phase follows the complex chemokine and cytokine responses modulating inflammation, secondary injury, and attempts for repair, which span between seven days to two weeks [37]. In this study, the focus of observation was on the acute phase of injury due to extensive publications on therapeutic animal trials focusing on treatment one-week following injury induction. Previous studies have shown that this acute injury phase is the ideal therapeutic target period [38]. Understanding the immediate, direct effects of injury using the balloon compression model is crucial for establishing a benchmark to evaluate the efficacy of therapeutic interventions in the acute phase of spinal cord injury.

In the acute phase of SCI, edema, hemorrhage, and inflammation are the primary microscopic findings observed in humans [39]. In our study, H&E staining revealed significant intramedullary hemorrhage and inflammation at the injury site. Hemorrhage results from micro-injuries to blood vessels following the initial trauma, triggering blood aggregation to stop the bleeding [39,40]. However, the aggregation process often lags the hemorrhaging, potentially inducing necrosis or apoptosis in glial cells, which can be observed through H&E staining and vacuolization in LFB staining [23]. As the initial injury mechanism damages surrounding tissues, an inflammatory response is activated to clear necrotic debris identified as foreign in the spinal cord [23,24]. The reason why edema was not observed remains unknown. However, further immunohistochemistry staining, such as the β-amyloid precursor protein (APP), can be utilized to indirectly detect edema of the axon [24-25]. Alisauskaite *et al*. [25] and Sptizbarth *et al*. [41] also found hemorrhage, inflammation, and necrosis in the subacute phase of the dog model. However, they also mentioned the findings of glial regeneration. The supra-lesional and infra-lesional samples also showed signs of inflammation and hemorrhage, although not to the extent of the intra-lesional samples, thus indicating the pathological pattern following balloon compression induction centralized around the initial area of contact of catheter inflation. The hemorrhage observed may be due to the compression and subsequent rupture of spinal arteries, leading to intramedullary extravasation.

Our findings using LFB staining revealed complete demyelination in intralesional samples in all dogs except for dog 4. Complete demyelination is used as an indication of structural damage [15]. The presence of complete structural intralesional samples shows the success of balloon compression in inducing spinal cord injury at the T10-T11 height. Observations of vacuole formation indicate the ongoing process of cellular apoptosis due to the initial injury. Vacuole formation in supra and infra lesional samples indicate that injury following balloon compression in the long term may not be limited to the intralesional area, which is supported by findings in samples from dog 4, as well as the observed hemorrhage and inflammation in H&E staining of supra and infra lesional samples.

## CONCLUSION

Our study demonstrates that balloon compression at the T10-T11 spinal level in dogs for six hours effectively induced spinal cord injury. This was evidenced by significant motor function impairment and complete demyelination observed in LFB-stained samples. The balloon compression model at the T10-T11 level, using an inflated volume of 1.0 ml, minimizes the risk of balloon rupture. A longer duration of compression ensures total paralysis in this model. Motor function remained impaired throughout the observational period, thus providing a platform for therapeutic interventions to be tested during the acute phase of SCI. Further research on large animal models for SCI study should be done to fill the gap between findings in small animal model studies and applications in the clinical setting.
